# Supplementary material for: MetaRibo-Seq measures translation in microbiomes
Source: Nat Commun. 2020 Jun 29;11:3268. doi: 10.1038/s41467-020-17081-z (PMC7324362; doi:10.1038/s41467-020-17081-z)
Supplement: Supplementary file 10 — Supplementary Data 7 [file 41467_2020_17081_MOESM10_ESM.zip › File2/Confidence_High_Taxonomy/29758_out.krona.html]

Javascript must be enabled to view this page.

members
magnitude
magnitudeUnassigned
count
unassigned
taxon
rank

29758\_out

153

153
superkingdom
2

976
phylum
153

class
200643
153

153

SRS077641\_contig\_number\_contig-100\_1745.117400SRS1041137\_contig\_number\_21077SRS1041145\_contig\_number\_15960SRS1055034\_contig\_number\_contig-100\_39929.39930
order
4
171549

52
815
family

816
genus
2

SRS016018\_contig\_number\_13702SRS1054716\_contig\_number\_10555
52


SRS076756\_contig\_number\_6892SRS148721\_contig\_number\_49888
2
28116
species


SRS144506\_contig\_number\_20933
1
817
species

15

SRS013521\_contig\_number\_9365SRS014412\_contig\_number\_15933SRS016267\_contig\_number\_contig-100\_3711.69455SRS017821\_contig\_number\_26281SRS019787\_contig\_number\_3102SRS042966\_contig\_number\_13061SRS046068\_contig\_number\_2356SRS049896\_contig\_number\_22354SRS051031\_contig\_number\_33196SRS077392\_contig\_number\_41404SRS078176\_contig\_number\_1138SRS100907\_contig\_number\_contig-100\_491.62933SRS1041142\_contig\_number\_5523SRS143148\_contig\_number\_21115SRS893327\_contig\_number\_7601
species
1262738

1

SRS021948\_contig\_number\_28102
species
2292941

species
1638779
30

SRS015369\_contig\_number\_6671SRS015960\_contig\_number\_12272SRS016056\_contig\_number\_3188SRS016989\_contig\_number\_11089SRS017103\_contig\_number\_25864SRS017745\_contig\_number\_contig-100\_7269.7270SRS018836\_contig\_number\_14256SRS018888\_contig\_number\_484SRS019910\_contig\_number\_17318SRS020622\_contig\_number\_7064SRS024331\_contig\_number\_20205SRS024435\_contig\_number\_33339SRS043841\_contig\_number\_9490SRS044535\_contig\_number\_18363SRS052027\_contig\_number\_535SRS053356\_contig\_number\_23948SRS053398\_contig\_number\_18693SRS056259\_contig\_number\_23398SRS064557\_contig\_number\_19952SRS075773\_contig\_number\_contig-100\_2645.196909SRS077231\_contig\_number\_19479SRS077335\_contig\_number\_7728SRS098644\_contig\_number\_13530SRS1041090\_contig\_number\_5735SRS1041130\_contig\_number\_762SRS104975\_contig\_number\_24103SRS143895\_contig\_number\_41906SRS146812\_contig\_number\_54184SRS148196\_contig\_number\_18116SRS893300\_contig\_number\_1494

1

SRS012849\_contig\_number\_27980
species
821

2005525
family
4

1
195950
genus

species
2293248
1

SRS048164\_contig\_number\_17900


SRS013800\_contig\_number\_12873SRS020328\_contig\_number\_32297
3
375288
genus
2

species
46503
1

SRS148091\_contig\_number\_10271

3
171550
family

3
239759
genus

2

SRS046369\_contig\_number\_1336SRS065504\_contig\_number\_32729
species
214856


SRS042628\_contig\_number\_36407
1
28117
species

family
171552
2

2
genus
838


SRS1041140\_contig\_number\_21071
1
165179
species


SRS019445\_contig\_number\_8669
1
59823
species


SRS011302\_contig\_number\_26522SRS018133\_contig\_number\_13517SRS075821\_contig\_number\_20474SRS893341\_contig\_number\_685
4
1897039
species

171551
family
1

1952625
species

SRS011084\_contig\_number\_28873
1

2005519
family
79


SRS013951\_contig\_number\_contig-100\_8706.57065SRS049402\_contig\_number\_14688SRS1041143\_contig\_number\_3152SRS140492\_contig\_number\_27667SRS893292\_contig\_number\_contig-100\_624.625SRS893295\_contig\_number\_8515
79
397864
genus
6

species
487174
47

SRS015663\_contig\_number\_24478SRS015694\_contig\_number\_7220SRS015794\_contig\_number\_8360SRS017521\_contig\_number\_42573SRS017701\_contig\_number\_12212SRS018351\_contig\_number\_17052SRS018936\_contig\_number\_543SRS019161\_contig\_number\_33160SRS019381\_contig\_number\_12937SRS019968\_contig\_number\_contig-100\_136.104130SRS022524\_contig\_number\_8802SRS043768\_contig\_number\_25074SRS050520\_contig\_number\_287SRS050752\_contig\_number\_22067SRS052697\_contig\_number\_33526SRS053649\_contig\_number\_16947SRS054059\_contig\_number\_4788SRS054352\_contig\_number\_23440SRS055017\_contig\_number\_29246SRS056273\_contig\_number\_25726SRS062654\_contig\_number\_8869SRS063040\_contig\_number\_29876SRS063370\_contig\_number\_9842SRS065176\_contig\_number\_14014SRS075078\_contig\_number\_31588SRS075341\_contig\_number\_24875SRS077294\_contig\_number\_6053SRS077552\_contig\_number\_11575SRS078242\_contig\_number\_23578SRS078419\_contig\_number\_10270SRS097920\_contig\_number\_16227SRS098571\_contig\_number\_13303SRS103987\_contig\_number\_2935SRS1041138\_contig\_number\_14086SRS143070\_contig\_number\_936SRS144603\_contig\_number\_12411SRS145497\_contig\_number\_11439SRS147271\_contig\_number\_11961SRS147557\_contig\_number\_31555SRS147614\_contig\_number\_2876SRS149181\_contig\_number\_19826SRS893252\_contig\_number\_13423SRS893279\_contig\_number\_4040SRS893342\_contig\_number\_6042SRS893358\_contig\_number\_1681SRS893378\_contig\_number\_14409SRS893383\_contig\_number\_5141


SRS016954\_contig\_number\_23251SRS017307\_contig\_number\_21998SRS020233\_contig\_number\_25661SRS021484\_contig\_number\_3849SRS022609\_contig\_number\_41873SRS024132\_contig\_number\_41751SRS024625\_contig\_number\_10494SRS045826\_contig\_number\_20662SRS047433\_contig\_number\_18787SRS049712\_contig\_number\_15979SRS049773\_contig\_number\_17825SRS050941\_contig\_number\_17725SRS058770\_contig\_number\_15314SRS076976\_contig\_number\_17132SRS077127\_contig\_number\_5501SRS105082\_contig\_number\_14777SRS1055056\_contig\_number\_1999SRS1055076\_contig\_number\_10522SRS143523\_contig\_number\_21900SRS143876\_contig\_number\_26891SRS144183\_contig\_number\_27497SRS148511\_contig\_number\_13266SRS148784\_contig\_number\_contig-100\_454.108766SRS150029\_contig\_number\_17617SRS893170\_contig\_number\_5895SRS893382\_contig\_number\_contig-100\_8470.8471
26
2033407
species

family
1853231
4

3
283168
genus

species
28118
3

SRS019397\_contig\_number\_9401SRS064757\_contig\_number\_5269SRS140513\_contig\_number\_1032

genus
574697
1

species
544645
1

SRS049995\_contig\_number\_16765
